# Noninvasive electrocardiography monitoring for very early recurrence predicts long‐term outcome in patients after atrial fibrillation ablation

**DOI:** 10.1111/anec.12785

**Published:** 2020-06-25

**Authors:** De‐yan Yang, Zhong‐wei Cheng, Yong‐tai Liu, Peng Gao, Tai‐bo Chen, Hua Deng, Kang‐an Cheng, Jing‐bo Fan, Quan Fang

**Affiliations:** ^1^ Department of Cardiology Peking Union Medical College Hospital Chinese Academy of Medical Sciences & Peking Union Medical College Beijing China

**Keywords:** atrial fibrillation, catheter ablation, noninvasive electrocardiography, very early recurrence

## Abstract

**Background:**

Atrial fibrillation (AF) is the most common sustained arrhythmia, and catheter ablation has been shown to be a highly effective treatment for patients with symptomatic AF. Very early recurrence (VER) of AF within 7 days after catheter ablation is common, but the clinical significance of VER remains unclear. We have examined the usefulness of the noninvasive electrocardiography monitor for the detection of VER and the relationship between VER and late recurrence (LR).

**Methods:**

Eighty‐eight patients with paroxysmal or persistent atrial fibrillation were retrospectively included. All patients underwent primary catheter ablation at a large general hospital between March 2016 and August 2018. All patients were followed up in atrial fibrillation clinic at an interval of every 3 months for late recurrence of AF. VER was evaluated by one‐lead continuous noninvasive electrocardiography monitoring device for 7 days after ablation. The association between VER and LR was analyzed by univariate and multivariate Cox regression model.

**Results:**

Mean age was 62.9 ± 9.7 years, and 39.8% were female. Thirty‐two patients (36.4%) experienced VER. After a mean follow‐up of 539.36 ± 211.66 days, 17 patients (19.3%) experienced LR. Multivariate Cox regression analysis revealed VER was an independent predictor of LR: HR 3.6 (95% CI, 1.2–10.8), *p* = .020. In addition, diabetes was also associated with LR of atrial fibrillation.

**Conclusions:**

Noninvasive electrocardiography monitoring was a useful tool for detecting VER and VER after catheter ablation was associated with LR.

## 1 INTRODUCTION

Atrial fibrillation (AF) is the most common sustained arrhythmia, and catheter ablation has been shown to be a highly effective treatment for patients with symptomatic AF (Calkins et al., 2018; Kirchhof et al., 2016). Early recurrences after AF ablation within 3 months are common, and substantial proportion of early recurrences spontaneously relieve beyond 3 months (Miyazaki et al., 2015; Nalliah et al., 2015; Pokushalov et al., 2012). Hence, early reablation is not recommended within 3 months blanking period and early recurrences are not considered as ablation failure (Calkins et al., 2018; Kirchhof et al., 2016). However, recent studies indicated that early recurrences were associated with late recurrences (LR) (Miyazaki et al., 2015; Nalliah et al., 2015; Willems et al., 2016) and early detection and early reablation might have potential benefits (Lellouche et al., 2008; Pokushalov et al., 2011).

Most of early recurrences occur within 7 days after ablation (Oral et al., 2002), but studies focusing on the association between very early recurrences (VER) within 7 days postablation and LR are scarce and the clinical significance of VER remains unclear (Arya et al., 2010; Xue et al., 2017).

In this study, we sought to investigate the relationship between VER detected by one‐lead noninvasive electrocardiography monitoring device and LR after AF catheter ablation.

## 2 METHODS

### 2.1 Patient population

Consecutive patients with symptomatic AF underwent initial catheter ablation between March 1, 2016, and August 30, 2018 in Peking Union Medical College Hospital were retrospectively screened. The indications for catheter ablation for AF were in compliance with the most recent guideline (Calkins et al., 2018; Kirchhof et al., 2016). All patients provided written informed consent, and the procedures of this study were approved by the Institutional Review Board of Peking Union Medical Hospital. AF was classified according to current guideline (Kirchhof et al., 2016). Patients were excluded if they aged < 18, had contraindications for anticoagulation, heart failure (NYHA III or IV), implanted pacemaker or implantable cardioverter–defibrillator, or expected survival less than 1 year. All patients were encouraged to receive 7 days noninvasive electrocardiography monitoring after procedure. Patient clinical characteristics, medical history, echocardiographic features, and medication were recorded. CHA_2_DS_2_‐VASc score was calculated for each patient.

### 2.2 Patient treatment and management

Patients without left atrial thrombus after transesophageal echocardiography screening underwent ablation under mild sedation with midazolam. After a transseptal puncture, pulmonary vein isolation (PVI) was performed with a 3.5‐mm ablation catheter with an externally irrigated tip (NaviStar ThermoCool SmartTouch, Biosense Webster, Inc.) under the guidance of a three‐dimensional electroanatomical mapping system (CARTO 3, Biosense Webster, Inc.) by delivering radiofrequency energy at a maximum temperature of 43°C and a maximum power of 35W for 30s at a flow rate of 17–30 ml/min. During the procedure, systemic anticoagulation was achieved with intravenous heparin titrated to maintain an activated clotting time of ≥ 350 s. Cardioversion of sustained AF was achieved by intravenous ibutilide or electrical cardioversion. The endpoint of the PVI was achievement of bidirectional conduction block between the left atrium and the pulmonary veins at least 30 min after initial isolation. Following PVI, reproducibly induced organized atrial tachyarrhythmias (atrial tachycardia or atrial flutter) were also targeted as guided by activation and/or entrainment mapping, and left atrial linear ablation was performed. Complete block along the cavotricuspid isthmus was created if common atrial flutter was detected before or during the procedure. Superior vein cava was isolated if premature atrial contraction or atrial tachycardia originating from superior vein cava was detected. Amiodarone or propafenone was prescribed for 1–3 months after the procedure, and oral anticoagulation drugs were continued for at least 2 months (Calkins et al., 2018).

### 2.3 Postablation noninvasive electrocardiography monitoring

Patients were encouraged to undergo continuous noninvasive electrocardiography (ECG) monitoring immediately (within 30 min) after ablation. A commercial available China Food and Drug Administration (CFDA) approved noninvasive ECG monitor (Ambulatory Electrocardiography System PE‐C, Prudence Medical Technologies, Shanghai, China) with adhesive electrodes (3M, Sao Paulo, Minnesota, USA) was attached on the prethoracic area of the patient. The device had a memory capable of recording ECG for up to 30 days using simulative one channel with 256 Hz sampling rate. Technical parameters including dynamic range (±5 mV), input impedance (>10 MΩ), common mode rejection (≥60 dB), gain accuracy (≤10%), gain stability (≤3% over a 24‐hr period), system noise (<50uVp‐v), and frequency response (0.67–55 Hz) were set as factory setting. Patients were planned to be monitored for 7 days (Arya et al., 2010; Xue et al., 2017) and returned the device to research assistant after 7 days monitoring. Patients received both verbal and written instructions on how to use the device. The researchers had no financial engagement with the ECG device manufacturer. The company was not involved in planning of the study, data analysis, or reporting. Data were exported and analyzed by software (Ambulatory Electrocardiography System PE‐C, Prudence Medical Technologies). Length of monitoring and ECG data quality were recorded. If the duration of monitoring was longer than 7 days, only data within 7 days were analyzed; while the duration of monitoring was less than 7 days, data analysis was based on the longest monitoring duration. The suggested atrial arrhythmias episodes were adjudicated by two experienced raters (Jing‐bo Fan and Zhong‐wei Cheng). Very early recurrence was defined as sustained AF or atrial flutter (AFL) or atrial tachycardia (AT) (lasting > 30 s) observed within 7 days postablation (Arya et al., 2010; Xue et al., 2017).

### 2.4 Follow‐up

Follow‐up visits in atrial fibrillation clinic consisted of clinical interviews, ECGs, and/or 24‐hr Holter monitoring were performed every 3 months. Late recurrence was defined if AF or atrial flutter or atrial tachycardia lasting longer than 30s documented by 24‐hr Holter or documented by ECG after the initial 3‐month blanking period postablation. The date of LR, the interval between initial ablation and LR, and the antiarrhythmia drugs during last visit were recorded.

### 2.5 Statistical analysis

Continuous data were expressed as the mean (± standard deviation [*SD*]) for normally distributed variables or as the median (interquartile range [IQR]) for non‐normally distributed variables, and were compared using Student's *t* test or Mann–Whitney U test, when appropriate. Categorical variables were presented as counts or proportions, and were compared using chi‐square test or Fisher's exact test. Baseline characteristics of patients who did and did not undergo noninvasive electrocardiography monitoring were compared. The univariate and multivariate Cox regression method was used to determine the predictors of LR. Variables were selected for univariate analysis according to medical knowledge and previous studies, and then, all variables with a *p*‐value <.1 in the univariate analyses were included in the multivariate Cox regression model (enter method). All *p*‐values were two‐sided, and the results were considered statistically significant at the level of *p* < .05. SPSS software (version 19.0, IBM) was used for all statistical analyses.

## 3 RESULTS

### 3.1 Patient characteristics

**FIGURE 1:**
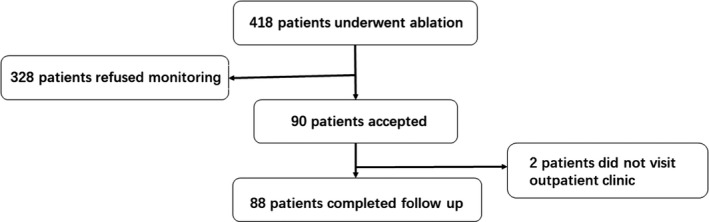
Enrolling flowchart. Four hundred eighteen patients with AF underwent ablation: 328 patients declined, and 90 patients accepted. Two patients were lost to follow‐up, and 88 patients were included in this study

A total of 418 consecutive adult patients with AF underwent initial catheter ablation: 328 patients declined postablation ECG monitoring because of difficulties for returning monitoring devices due to living far away from our hospital while 90 patients accepted. Baseline characteristics of patients who did and did not undergo noninvasive electrocardiography monitoring were similar except additional ablation which was more common among patients without 7 days monitoring (Table 1). Among 90 patients received noninvasive electrocardiography monitoring, eighty‐eight (97.8%) patients completed follow‐up and were included in this study (Figure 1). Baseline characteristics of 88 patients (35 female, mean age 62.9 ± 9.7 years) stratified by the late recurrence of AF are summarized in Table 2. AF was paroxysmal in 58 (65.9%) patients and persistent in 30 (34.1%) patients. Duration of AF was significantly longer in patients with LR, *p* = .009.

**Table 1:** Comparison of baseline characteristics between patients with and without noninvasive electrocardiography monitoring (*n* = 418)

Variable	All (*n* = 418)	Noninvasive electrocardiography monitoring		*p*‐value
		With (*n* = 90)	Without (*n* = 328)	
Age, years	62.9 ± 10.9	62.8 ± 9.6	62.9 ± 11.3	.961
Age ≥ 65 years	199 (47.6)	41 (45.6)	158 (48.2)	.721
Female	166 (39.7)	37 (41.1)	129 (39.3)	.808
BMI, kg/m^2^	25.6 ± 3.2	25.7 ± 3.5	25.6 ± 3.1	.980
Type of AF				
Paroxysmal	281 (67.2)	60 (66.7)	221 (67.4)	.900
Persistent	137 (32.8)	30 (33.3)	107 (32.6)	.900
Duration of AF, months	12.0 (2.0–53.0)	9.5 (2.8–45.0)	13.0 (2.0–57.0)	.308
Hypertension	248 (59.3)	55 (61.1)	193 (58.8)	.718
Diabetes	96 ( (23.0)	21 （23.3)	75 (22.9)	>.999
CAD	83 (19.9)	17 (18.9)	66 (20.1)	.882
VHD	7 (1.7)	2 (2.2)	5 (1.5)	.647
History of HF	15 (3.6)	3 (3.3)	12 (3.7)	>.999
Prior stroke/TIA	33 (7.9)	3 (3.3)	30 (9.1)	.079
LAD (mm)	41.1 ± 5.6	40.4 ± 6.2	41.3 ± 5.4	.162
LAD ≥ 40mm	258 (61.7)	53 (58.9)	205 (62.5)	.542
LVEF (%)	65.5 ± 9.5	66.5 ± 7.8	65.3 ± 9.9	.268
CHA_2_DS_2_‐VASc score	2.1 ± 1.5	2.0 ± 1.2	2.1 ± 1.5	.331
Additional ablation	111 (26.6)	16 (17.8)	95 (29.0)	.043
AAD at discharge	354 (84.7)	81 (90.0)	273 (83.2)	.137
Amiodarone	254 (60.8)	62 (68.9)	192 (58.5)	.088
Propafenone	100 (23.9)	19 (21.1)	81 (24.7)	.577

Note

Data are mean ± standard deviation or *n* (%) or median (interquartile range).

Abbreviation: AAD, antiarrhythmia drug; AF, atrial fibrillation; BMI, body mass index; CAD, coronary artery disease; HF, heart failure; LAD, left atrial dimension; LVEF, left ventricular ejection fraction; TIA, transient ischemic attack; VER, very early recurrence; VHD, valvular heart disease.

**Table 2:** Baseline characteristics of the study patients (*n* = 88)

Variable	All (*n* = 88)	Late recurrence		*p*‐value
		With (*n* = 17)	Without (*n* = 71)	
Age, years	62.9 ± 9.7	62.1 ± 8.9	63.0 ± 9.9	.726
Age ≥ 65 years	41 (46.6)	6 (35.3)	35 (49.3)	.418
Female	35 (39.8)	10 (58.8)	25 (35.2)	.099
BMI, kg/m^2^	25.7 ± 3.5	25.9 ± 2.4	25.7 ± 3.7	.801
Type of AF				
Paroxysmal	58 (65.9)	11 (64.7)	47 (66.2)	>.999
Persistent	30 (34.1)	6 (35.3)	24 (33.8)	>.999
Duration of AF, months	9.5 (2.3–42.0)	36.0 (4.5–120.0)	6.0 (1.0–24.0)	.009
Hypertension	54 (61.4)	11 (64.7)	43 (60.6)	>.999
Diabetes	21 (23.9)	7 (41.2)	14 (19.7)	.109
CAD	17 (19.3)	5 (29.4)	12 (16.9)	.305
VHD	2 (2.3)	0 (0.0)	2 (2.8)	>.999
History of HF	3 (3.4)	1 (5.9)	2 (2.8)	.479
Prior stroke/TIA	3 (3.4)	1 (5.9)	2 (2.8)	.479
LAD (mm)	40.5 ± 6.2	39.0 ± 5.7	40.9 ± 6.4	.272
LAD ≥ 40 mm	52 (59.1)	10 (58.8)	42 (59.2)	>.999
LVEF (%)	66.6 ± 7.9	64.4 ± 7.5	67.1 ± 7.9	.208
CHA_2_DS_2_‐VASc score	2.00 ± 1.3	2.3 ± 1.3	1.9 ± 1.2	.250
Additional ablation	14 (15.9)	4 (23.5)	10 (14.1)	.459
AAD at discharge	79 (89.8)	16 (94.1)	63 (88.7)	>.999
Amiodarone	60 (68.2)	12 (70.6)	48 (67.6)	>.999
Propafenone	19 (21.6)	4 (23.5)	15 (21.1)	>.999
VER	32 (36.4)	9 (52.9)	23 (32.4)	.160

Note

Data are mean ± standard deviation or *n* (%) or median (interquartile range).

Abbreviation: AAD, antiarrhythmia drug; AF, atrial fibrillation; BMI, body mass index; CAD, coronary artery disease; HF, heart failure; LAD, left atrial dimension; LVEF, left ventricular ejection fraction; TIA, transient ischemic attack; VER, very early recurrence; VHD, valvular heart disease.

### 3.2 Very early recurrence

Monitoring duration ranges from 24 hr to 388 hr with median of 169 hr (IQR 159–209), and the monitoring duration of 29 (33.0%) patients was less than 7 days. Analyzable data range from 23 hr to 385 hr with median of 161 hr (IQR 127–196), and the percentage of analyzable data range from 57.1% to 100.0% with median of 96.0% (IQR 86.7–99.1). Twenty‐five patients had AF only, 4 patients had AT only, and 1 patient had AFL only. One patient had both AF and AT, and another one experienced both AF, AT, and AFL. Hence, there were a total of 32 (36.4%) patients experienced VER.

### 3.3 Follow‐up

During a mean follow‐up of 539.4 ± 211.7 days, antiarrhythmic drugs (AAD) were discontinued in 81.8% of patients. Seventeen (19.3%) patients experienced LR consisted of paroxysmal AF, persistent AF, persistent atrial flutter, and paroxysmal atrial tachycardia in 12, 2, 2, and 1 patients, respectively. Among 32 patients with VER, 9 (28.1%) patients experienced LR while 8 (14.3%) patients experienced LR among 56 patients without VER. Among 17 patients with LR, 6 patients underwent reablation and 6 patients were treated with AAD while 5 patients did not receive any AAD because of mild symptom.

**FIGURE 2:**
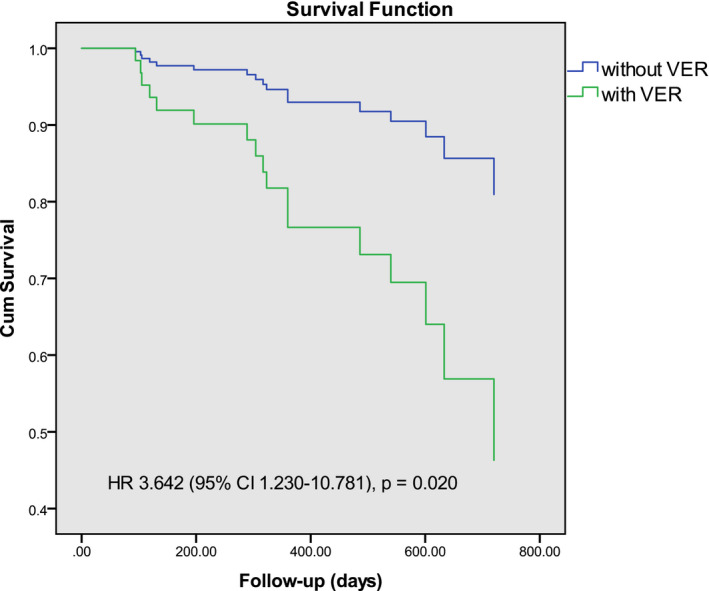
Multivariate Cox regression survival curve for freedom from late recurrence in patients with VER and those without VER

Diabetes (HR 3.35, 95% CI, 1.19–9.42; *p* = .02) and VER (HR 3.64, 95% CI, 1.23–10.78; *p* = .02) were independent predictors of LR in the multivariate Cox regression model (Table 3 and Figure 2).

**Table 3:** Univariate and multivariable factors associated with late recurrence

Variable	Univariate			Multivariate		
	HR	95% CI	*p*	HR	95% CI	*p*
Female	2.330	0.886–6.129	.086	2.492	0.905–6.860	.077
Age	1.000	0.953–1.049	.990			
Age ≥ 65 years	0.724	0.267–1.963	.526			
BMI	1.009	0.874–1.164	.906			
Persistent AF	1.047	0.387–2.835	.928			
Duration of AF	1.008	1.001–1.014	.022	1.004	0.998–1.011	.194
Hypertension	1.169	0.432–3.164	.759			
Diabetes	2.466	0.935–6.500	.068	3.354	1.194–9.422	.022
CAD	2.073	0.727–5.905	.172			
LAD	0.954	0.883–1.030	.225			
LAD ≥ 40 mm	0.935	0.354–2.470	.891			
Additional ablation	0.690	0.224–2.128	.519			
VER	2.371	0.910–6.179	.077	3.642	1.230–10.781	.020

Abbreviation: AF, atrial fibrillation; BMI, body mass index; CAD, coronary artery disease; LAD, eft atrial dimension; VER, very early recurrence.

## 4 DISCUSSION

The main findings of our study are threefold: (a) More than one third of patients experienced VER within 7 days postparoxysmal or persistent atrial fibrillation ablation; (b) A simple noninvasive short‐term ECG monitoring device was useful to detect VER; and (c) VER was an independent predictor of LR, and LR for patients with VER was nearly four times the risk of those without VER.

A retrospective study including paroxysmal and persistent AF had found that more than half of patients experienced VER after ablation during 7 days Holter recording (Arya et al., 2010) and a recent prospective study (Xue et al., 2017) shown that nearly one third of patient developed VER after AF ablation. Our study is consisted with these results which demonstrated that VER is common.

Transient inflammatory response contributed greatly to early recurrence after ablation (Lim et al., 2014), and early recurrence may subside spontaneously in a considerable proportion of patients during follow‐up. Besides inflammatory response, pulmonary vein reconnection also plays an important role in early recurrence (Das et al., 2015). A prospective randomized study indicated that early reablation within 3‐month postinitial ablation might reduce the risk of late recurrence (Pokushalov et al., 2011). Furthermore, early recurrence AF burden higher than 4.5% at 2 months postablation was an independent predictor of late recurrence (Pokushalov et al., 2012) and the later the early recurrence occurred the higher risk for late recurrence (Alipour et al., 2017). However, invasive implanted monitoring device and long‐term frequent Holter monitoring were needed for the determination of AF burden and the early recurrence timing (Alipour et al., 2017; Pokushalov et al., 2012). Until now, implantable subcutaneous ECG monitor was not widely available in China and most postablation patients left hospital and returned to their local cities which result in difficulties for intensively long‐term monitoring. A short‐term noninvasive ECG monitoring might be an alternative method for very early recurrence detection and helped to screen patients who were at high risk of late recurrence and should be under individual follow‐up strategy.

In Xue's study (Xue et al., 2017), VER was an independent predictor of long‐term recurrence and our study is in line with this study which VER was screened by continuous ECG monitoring and patients had to remained hospitalized for at least 7 days. However, VER in the present study was evaluated by one‐lead ECG device taken home by patients which might improve patient adherence.

### 4.1 Study limitation

This study was limited by its retrospective nature. The series was neither consecutive nor large because of majority of patients refusing the monitoring which might cause biases. The mechanisms of VER and LR in the present study were not investigated. Later recurrence evaluated by ECGs and/or 24‐hr Holter might be underestimated.

## 5 CONCLUSIONS

Very early recurrences within 7 days after atrial fibrillation ablation were common and could be detected by the noninvasive monitoring method. Very early recurrence was associated with late recurrence. Short‐term ECG monitoring should be performed after AF ablation, and electrophysiologists should give close observation to patients with VER.

## CONFLICT OF INTEREST

We declare that we have no financial and personal relationships with other people or organizations that can inappropriately influence our work.

## ETHICAL APPROVAL

All patients provided written informed consent, and the procedures of this study were approved by the Institutional Review Board of Peking Union Medical Hospital.
